# Latent classes of energy and nutrient intake and their associations with oxidative stress in rural older adults: a cross-sectional study

**DOI:** 10.3389/fnut.2025.1694444

**Published:** 2025-12-08

**Authors:** Meiman Li, Weijuan Kong, Ting Jiang, Yanhua Ning

**Affiliations:** 1 Department of Master’s Training Station, General Hospital of Ningxia Medical University, Yinchuan, China; 2 School of Nursing, Ningxia Medical University, Yinchuan, China

**Keywords:** energy, nutrient, oxidative stress, latent class analysis, older adults

## Abstract

**Background:**

Research indicates that diet correlates with oxidative stress; however, the influence of specific energy and nutrient classes has been scarcely studied. This study aimed to investigate the categories of energy and nutrient intake among rural older adults through a latent class analysis and to explore the association between each category and biomarkers of oxidative stress.

**Methods:**

It is a cross-sectional study. Dietary information was obtained from 3 days of 24-h dietary diaries. Levels of oxidative stress markers were measured from fasting venous blood samples. A latent class analysis was used to analyze energy and nutrient intake classes. Analysis of variance and *post-hoc* comparisons were used to examine the relationships between energy and nutrient classes and oxidative stress.

**Results:**

This analysis was performed on 376 adults aged 65 years and older. The latent class analysis found that energy and nutrient intake could be divided into three classes: over-adequate nutrition—high energy (*n* = 141, 37.50% of the total samples), adequate nutrition—low in energy and protein (*n* = 69, 18.35% of the total samples), and inadequate nutrition (*n* = 166, 44.15% of the total samples). 8-iso-PGF2α (*F* = 6.004, *p =* 0.003) and superoxide dismutase (SOD) (*F* = 5.559, *p* = 0.004) are associated with different energy and nutrient latent classes.

**Conclusion:**

This study explores how classes of energy and nutrients relate to oxidative stress in rural older adults. The findings provide a basis for future research on oxidative stress and nutrition in the elderly.

## Background

Population aging is a global challenge, accompanied by a rising prevalence of chronic diseases such as stroke, heart disease, and diabetes (1). A suboptimal diet and nutrition are considered important factors in the development of chronic diseases and mortality (2). Diet has been proved to be a modifiable behavior related to health (3).

Older adults are the most susceptible group to malnutrition (4). According to the World Health Organization (WHO) (5), malnutrition refers to deficiencies, excesses, or imbalances in a person’s intake of energy and/or nutrients. Current estimates suggest that approximately a quarter of older adults aged 65 years and above are malnourished or at risk of malnutrition (6). It has been reported that older adults are at risk of deficient or suboptimal micronutrient status, which leads to frailty and readmission (7, 8), and they are also at risk of overconsumption of macronutrients, which can lead to obesity and non-communicable diseases (9).

Malnutrition and oxidative stress may interact (10). Oxidative stress is generally described as a situation of imbalance in which excessive levels of oxygen free radicals or reactive oxygen species are present in the body in the face of inadequate availability of the relevant neutralizing substances—antioxidants (11). Oxidative stress is measured by malondialdehyde (MDA), 8-iso-prostaglandin F2α (8-iso-PGF2α), superoxide dismutase (SOD), and the total antioxidant capacity (T-AOC) (12–14). Oxidative stress is associated with the occurrence and development of many chronic diseases (15–18). Previous studies have reported that DNA, lipids, and protein peroxidation products are associated with oxidative stress in humans (19). In a review article, it is pointed out that oxidative stress is a critical factor in metabolic disorders associated with high-carbohydrate and animal-based protein diets and excessive fat consumption (20). Vitamin deficiency in the diet will have a damaging effect on the antioxidant defense system (21). Vitamin D supplementation may improve metabolic variables, reducing oxidative stress and cardiovascular disease outcomes for certain risk groups (22). Low folate and vitamin B_12_ levels are associated with increased oxidative stress in chronic pancreatitis patients (23). In a rat model of moderate environmental human exposure to cadmium, researchers found that zinc has a protective effect against the disruption of the oxidative/antioxidative balance (24).

Latent class analysis (LCA) has been widely used to classify dietary behaviors (25–27). Latent class analysis (LCA), a probabilistic modeling algorithm that allows clustering of data and statistical inference, and the unobserved, or “latent,” groups are inferred from patterns of the observed variables or “indicators” used in the modeling, allows investigators to determine if unmeasured or unobserved groups exist within a population (28, 29).

Despite concerns over the relationship between diet and oxidative stress in the older adult population, there is currently limited research in this vulnerable population group in Asian countries. To date, no studies have looked at the latent class of energy and nutrient intake and its association with oxidative stress in rural older adults aged 65 years and above in China. We hypothesized that the latent class of energy, macronutrients, and vitamins with antioxidant properties may influence oxidative stress levels in rural older adults. Therefore, this study aimed to examine patterns of energy and nutrient intake in older adults using latent class analysis and to explore the relationship between each class and biomarkers of oxidative stress.

## Methods

### Study design

This cross-sectional study was conducted via on-site questionnaire surveys using the convenient sampling method. First, we selected three rural community health stations in Yinchuan City and Wuzhong City, Ningxia, China. Second, we contacted community workers and conducted lectures on nutritional knowledge for older residents in the above community health stations. Third, potential participants were provided with informational documents, and they were given time to consider their participation. Written informed consent was obtained from all participants before the survey. The study was conducted between April and August 2021.

### Participants

The inclusion criteria for participants were older people aged 65 years and above who have resided in the rural community of Ningxia for more than 1 year and voluntarily participated in this study. The exclusion criteria for participants were as follows: (1) individuals with cognitive impairment, language disorders, and hearing impairments; (2) individuals with acute illnesses, acute exacerbations of chronic diseases, severe cardiorespiratory dysfunction, hepatic or renal insufficiency, end-stage diseases, or other conditions that may cause oxidative stress damage; and (3) individuals who have used immunosuppressants, vitamin C preparations, vitamin E preparations, or other agents that may affect indicator measurements within the past 3 months.

According to the standard deviation and allowable error of each oxidative stress index (30–33), PASS software was used to calculate the required sample size of each oxidative stress index (Table 1). The maximum sample size, calculated by selecting four indicators, represents the sample size for this study. Therefore, the sample size for this study is at least 350 participants.

**Table 1:** Sample sizes required for different oxidative stress indexes.

Sampling survey parameters	8-iso-PGF2α (pg/mL)	MDA (nmol/mg)	SOD (U/mL)	T-AOC (mmol/L)
Standard deviation	151.40	1.90	21.40	0.26
Allowable error	20.50	0.20	2.47	0.03
Sample size	212	350	291	291

### Measurements

All investigators completed a structured training program. We first explained the relevant precautions of collecting data for investigators, after which investigators conducted mock investigations in pairs. Blood collection was conducted by qualified nursing staff from the community health service station. The laboratory examinations were conducted independently under the guidance of a qualified technician.

Baseline demographics of the older adults contained items such as age, gender (male and female), education (uneducated, primary school, and junior high school and above), monthly income (below 1,000, 1,000 to 2000, and 2001 and above), marital status (married, unmarried/divorced/widowed), and employment (physical labor, mental labor, and unemployment).

Nutrition data were collected using 3-d 24-h dietary records. The investigators were trained prior to the survey, and a dietary model was used to establish a unified standard for food weights and the conversion methods. During the dietary survey, participants self-described their intake during breakfast, lunch, dinner, and snacks. Then, investigators combined the dietary models and standard portion photos of food with “hands” to accurately evaluate dietary intake. After the investigation is completed, verification should be conducted on the same day to avoid any omissions. The nutrition calculator developed by the Institute of Nutrition and Food Safety of the Chinese Center for Disease Control and Prevention and Beijing Feihua Communication Technology Co., Ltd. was used to input the 3-d 24-h dietary information; it output the energy and nutrient intakes of each participant. Energy, macronutrients, and vitamins were compared with the antioxidant function of participants with the recommended intake in the Dietary Guide for Elderly Adults (34).

Refer to relevant experimental methods (35). Participants were told to fast for 10 h. The participants’ venous blood was collected by a hygienically qualified nurse. After the blood specimen was placed at room temperature for 2 h, it was centrifuged at 3000 r/min for 10 min, and the supernatant was extracted. The supernatant was stored in a −80 °C refrigerator for later use. The concentrations of MDA, 8-iso-PGF2α, SOD, and T-AOC in the blood were determined by the following methods, respectively:

MDA: the thiobarbituric acid colorimetric method (Nanjing Jiancheng Bioengineering Institute); 8-iso-PGF2α: enzyme-linked immunosorbent assay (Elabscience Biotechnology Co., Ltd.); SOD: water-soluble tetrazole salt colorimetric method (Nanjing Jiancheng Bioengineering Institute); and T-AOC: chemiluminescence method (Nanjing Jiancheng Bioengineering Institute) (36, 37). The instruments, equipment used, and laboratory test items are listed in Supplementary Material A.

### Statistical analysis

#### Latent class analysis (LCA)

Mplus vision 8.3 was used for latent class analysis to examine the number of unobserved classes (the latent class of energy and nutrient intakes), describe the characteristics of the classes, and estimate the probabilities of class memberships for each individual (38). A latent class analysis (LCA) was performed to identify distinct homogeneous groups (latent classes) from categorical multivariate data in the case of this study (39). The LCA results revealed specific groups of energy and nutrient intakes present in the sample. The analysis included data for meeting grouping criteria for (1) energy, (2) protein, (3) fat, (4) carbohydrate, (5) vitamin A, (6) vitamin C, and (7) vitamin E. Five models with 1–5 classes were tested (analysis code can be found in Supplementary Material B), and the model selection was based on the results of a number of fit criteria (40): models with low values for the Akaike Information Criterion (AIC) and the Bayesian Information Criterion (BIC) indicated superior model fit among competing models. The entropy value indicated the distinctiveness of the latent classes when compared to one another, and values closer to one suggest clear classification. In addition, the Lo–Mendell–Rubin-adjusted likelihood ratio test (LMR-A) and parametric bootstrapped likelihood ratio test (BLRT) compared a *k*-class model to a *k*−1 model, where *k* is the number of latent classes. If the probability of *p*-value is <0.05, the *k*-class model is considered superior.

#### Subsequent analysis

Statistical analyses of latent class results and oxidative stress biomarkers were conducted using IBM SPSS Statistics 26.0 for Windows. All tests were two-sided with a *p-value* of 0.05. The means and standard deviations were used to describe the continuous variables. The counts and percentages were used to describe the categorical variables. Analysis of variance (ANOVA) was used to compare the differences between different groups of nutrient intake and oxidative stress biomarkers of the participants. For equal variances, the LSD *post-hoc* multiple comparison method was used; for unequal variances, Tamhane’s T2 *post-hoc* multiple comparison method was used.

## Results

### Sociodemographic and dietary characteristics

A total of 376 participants were included in this study. Sociodemographic characteristics and dietary characteristics of the older adults are presented in Table 2. Their average age was 72.06 years (SD = 5.95). There were slightly fewer males (47.34%) than females (52.66%). The proportion of older adults who were married was 77.93%; 22.07% were unmarried, divorced, or widowed. Approximately half (54.26%) of the older adults had uneducated, 26.86% of older adults completed primary school education, and the remaining had a junior high school and above degree. Referring to the Dietary Guide for Elderly Adults, when grouping the nutrient intake of older residents based on the reference intake (See in Supplemental material C), we found that insufficient energy and nutrient intake was common among the majority of the surveyed population.

**Table 2:** Sociodemographic and dietary characteristics of older adults (*n* = 376).

Variable			*n*	%
Gender	Male		178	47.34
	Female		198	52.66
Marital status	Married		293	77.93
	Unmarried/divorced/widowed		83	22.07
Education	Uneducated		204	54.26
	Primary school		101	26.86
	Junior high school and above		71	18.88
Monthly income	Below 1,000		273	72.61
	1,000–2000		44	11.70
	2001 and above		59	15.69
Employment	Physical labor		297	78.99
	Mental labor		51	13.56
	Unemployment		28	7.45
Energy and nutrient intakes	Energy	Insufficiency	154	40.96
		Moderateness	132	35.11
		Excessiveness	90	23.94
	Protein	Insufficiency	171	45.48
		Moderateness	112	29.79
		Excessiveness	93	24.73
	Fat	Insufficiency	313	83.24
		Moderateness	40	10.64
		Excessiveness	23	6.12
	Carbohydrate	Insufficiency	252	67.02
		Moderateness	94	25.00
		Excessiveness	30	7.98
	Vitamin A	Insufficiency	306	81.38
		Moderateness	53	14.10
		Excessiveness	17	4.52
	Vitamin C	Insufficiency	221	58.78
		Moderateness	94	25.00
		Excessiveness	61	16.22
	Vitamin E	Insufficiency	215	57.18
		Moderateness	78	20.74
		Excessiveness	83	22.07

### Oxidative stress results

The values and ranges of oxidative stress biomarkers among the participants are shown in Table 3.

**Table 3:** Levels of oxidative stress markers in the participants (*n* = 376).

Biomarkers of oxidative stress	Range	$\bar{X}$ ± S
MDA (nmol/mg)	1.58 ~ 11.29	4.82 ± 1.74
8-iso-PGF2α (pg/mL)	247.59 ~ 1788.34	782.13 ± 245.82
SOD (U/mL)	9.21 ~ 72.12	38.59 ± 10.69
T-AOC (mmol/L)	0.60 ~ 1.30	1.00 ± 0.15

T-AOC, alternate total antioxidant capacity; 8-iso-PGF2α, 8-iso-prostaglandin F2α; MDA, malondialdehyde; SOD, superoxide dismutase.

### Latent class model selection

The LCA model fit statistics are presented in Table 4, which presents the fitting results of the five models. In the latent class model, the main statistical indicators include *LL*, *AIC*, *BIC*, *aBIC*, *Entropy*, *LMR LR* (*P*), and *BLRT* (*P*). From the table, it can be observed that as the number of categories increases, the values of *LL*, *AIC*, *BIC*, and *aBIC* tend to decline. When the number of categories is 2, *AIC*, *BIC*, and *aBIC* are larger, and entropy is smaller. Therefore, category 2 is excluded. When the number of categories is 5, *AIC*, *BIC*, and *aBIC* are smaller, and LMR is not significant. Therefore, category 5 is excluded. Compared with three categories, four categories have a smaller *AIC* and *aBIC* but a larger *BIC* and a smaller *LL*. After a comprehensive comparison, category 3 is chosen.

**Table 4:** Results of the LCA—fit statistics (*n* = 376).

Model (numbers of latent classes)	*LL*	*AIC*	*BIC*	*aBIC*	*Entropy*	*LMR LR*	*BLRT*
						*p*-Values	*p*-Values
1	−2269.384	4566.768	3874.935	4577.363	–	–	–
2	−1851.488	3760.977	4431.29	3782.925	0.899	<0.001	<0.001
**3**	**−1735.821**	**3559.641**	**3732.543**	**3592.942**	**0.932**	**<0.001**	**<0.001**
4	−1692.886	3503.772	3735.618	3548.426	0.951	<0.001	<0.001
5	−1664.006	3476.011	3766.801	3532.018	0.947	0.684	<0.001

LL, Loglikelihood; AIC, Akaike Information Criterion; BIC, Bayesian Information Criterion; aBIC, Ajusted BIC; LMR LR, Vuong-Lo–Mendell–Rubin Likelihood Ratio Test; BLRT, Parametric Bootstrapped Likelihood Ratio Test. Class chosen is shown in bold.

**Figure 1:**
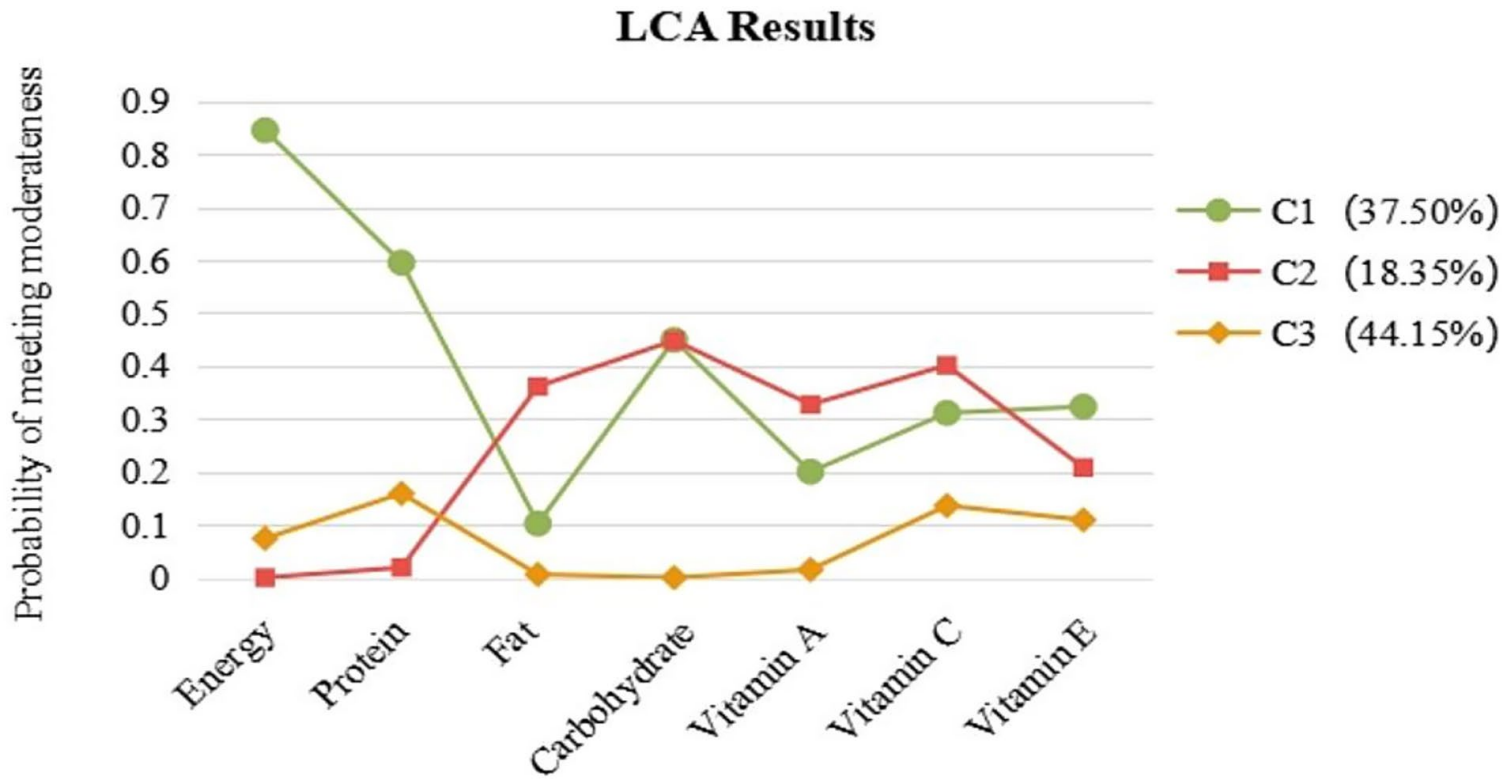
The class-profile figure. Percentages of most likely class belonging provided in brackets.

The class profile figure is shown in Figure 1. The first class (C 1: *n* = 141, 37.50% of the total samples) is characterized by an imbalance in energy and nutrient intake, with the probability of energy and protein consumption being significantly higher than that of other nutrients. We refer to the first class as “Over-adequate Nutrition—High Energy.” The second class (C 2: *n* = 69, 18.35% of the total samples) is characterized by sufficient and balanced nutritional substances other than energy and protein. Therefore, we refer to the second class as “Adequate Nutrition—Low in Energy and Protein.” The third class (C 3: *n* = 166, 44.15% of the total samples) is characterized by low energy intake and significant differences in the intake of various nutrients. We refer to the third class as “Inadequate Nutrition.”

### Oxidative stress across the latent class of energy and nutrient intakes

Table 5 shows the results of ANOVA and post-hoc comparisons. There were significant differences in means of 8-iso-PGF2α and SOD (*p* < 0.05) among the latent classes of energy and nutrient intake. However, there was no significant association between MDA, T-AOC, and the latent class of energy and nutrient intakes. It can be seen from the post-test results that for 8-iso-PGF2α, class 1 valued significantly higher than class 2 and class 3. As for SOD, class 2 valued significantly higher than class 1 and class 3.

**Table 5:** Comparison of different classes of energy and nutrient intakes with oxidative stress biomarkers (*n* = 376, $\bar{X}$ ± S).

Oxidative stress biomarkers	Class			*F*	*p*	Difference between class
	C 1	C 2	C 3			
MDA (nmol/mg)	4.875 ± 1.617	4.728 ± 2.031	4.821 ± 1.714	0.166	0.847	–
8-iso-PGF2α (pg/mL)	834.594 ± 280.369	721.029 ± 211.650	762.957 ± 218.852	6.004^b^	0.003	C1 > C2, C1 > C3
SOD (U/mL)	36.820 ± 10.090	42.007 ± 11.272	38.679 ± 10.647	5.599^a^	0.004	C2 > C1, C2 > C3
T-AOC (mmol/L)	1.001 ± 0.143	0.976 ± 0.157	1.019 ± 0.146	2.131	0.120	–

a LSD *post-hoc* multiple comparison method.

b Tamhane’s T2 *post-hoc* multiple comparison method.

## Discussion

In the current study, we used the latent class analysis to examine classes of energy and nutrient intake in older adults and to explore the relationship between each class and biomarkers of oxidative stress. There is limited research in China on the impact of diet on oxidative stress levels in the body. To the best of our knowledge, this study is the first to use latent class analysis to determine the relationship between energy and nutrient intake and oxidative stress levels in older adults in rural areas.

Our results show that older adults’ energy and nutrient intakes are heterogeneous. We identified three categories of energy and nutrient intake. The proportion of the “Inadequate Nutrition” class was the highest. It is characterized by low energy intake and significant differences in the intake of various nutrients. This is similar to the findings of scholar Caifang et al. (41). Such older adults need to improve their nutritional intake. Nutrients such as vitamins, β-carotene, polyphenols, selenium, and zinc, which are considered natural antioxidants (42), should be added to the daily diet of older adults. The second-largest category was “Over-adequate Nutrition—High Energy,” which is characterized by an imbalance in energy and nutrient intake. The category with the lowest proportion was “Adequate Nutrition—Low Energy and Protein.” It is characterized by sufficient and balanced nutritional substances other than energy and protein. Long-term imbalance in dietary intake is bound to lead to imbalance in energy and nutrient intake. This survey shows that insufficient energy and nutrient intake was common among the majority of the surveyed population. A balanced nutrient intake is crucial for older adults. They should increase the intake of high-quality protein and micronutrients by consuming more fruits, vegetables, eggs, milk and dairy products, legumes, and legume products. We recommend that specialized knowledge in dietary intake and the role of supplements in achieving recommended intakes should be integrated into elderly care (43). Additionally, we should also consider strategies to assist older adults in following a sensible diet and sticking to it.

Among the four oxidative stress biomarkers, the values of 8-iso-PGF2α and SOD differ across the three latent classes. MDA and T-AOC reflect the oxidative stress state and the overall antioxidant status of the body. The MDA and T-AOC values in the three energy and nutrient intake categories studied were not significantly affected.

8-iso-PGF2α is a product formed from the lipid peroxidation of arachidonic acid in the body. The 8-iso-PGF2α is considered the most comprehensive and reliable biomarker for assessing DNA and lipid oxidative damage and is the gold standard for measuring oxidative stress in the body (44). The 8-iso-PGF2α level in the over-adequate nutrition—high energy class is higher than in the other two classes. This indicates that there is a significant correlation between the intake of specific nutrients and the level of 8-iso-PGF2α. The over-adequate nutrition—high energy class is characterized by a higher intake of energy, protein, and carbohydrates compared to fat and vitamins, and the intake of energy is far higher than that of other nutrients. Studies have shown that high-energy, high-protein, and high-carbohydrate diets are associated with oxidative stress in the body (45–47). An animal study indicates that high-energy diets and obesity induce oxidative stress in pigeons (48). A study has also revealed that energy balance is associated with a lower risk of chronic diseases (49). Therefore, an imbalance in energy intake may be related to the induction of oxidative stress. Existing studies have revealed that vitamins can reduce ROS levels in patients with chronic kidney disease and those with critical illness (50, 51). Both domestic and international studies have confirmed that vitamins and their supplements can effectively reduce oxidative stress levels in humans (52–54). For instance, ascorbic acid (vitamin C) has four -OH groups that can donate hydrogen to an oxidizing system (55). γ-tocopherol is the primary form of vitamin E in the diet and exhibits potent antioxidant properties (56). Taken together, a dietary pattern that includes adequate vitamins and maintains a balanced intake of energy, protein, carbohydrates, and fats may be a useful nutritional intervention for the secondary prevention of age-related diseases.

SOD is the most common marker of oxidative damage. It can eliminate hydrogen peroxide and oxygen produced by free radicals, thereby reducing oxidative stress damage to the body (12). The SOD level in the adequate nutrition—low in energy and protein class is higher than in the other two classes. This indicates that there is a significant correlation between the intake of specific nutrients and the level of SOD. This class is characterized by sufficient and balanced nutritional substances other than energy and protein. The specific nutrients in dietary regimens and their balance can significantly influence overall health and changes in risk factors, such as cholesterol levels and blood pressure (57). From our study, compared with over-adequate nutrition and inadequate nutrient intake, a diet with sufficient nutrient intakes and lower energy and protein intake within a reasonable range leads to higher SOD concentrations and less oxidative stress damage to the body. Therefore, adopting a diet that adheres to a balanced and healthy eating pattern is an important and safe alternative for disease prevention. For the elderly aged 60 years and above, it is necessary to carry out health education through all kinds of institutions at all levels. They can regularly distribute “Residents’ Dietary Pyramid” posters to residents for free and supervise their placement in prominent positions in the kitchen. This method not only helps residents better understand the content of the dietary pyramid but also reminds the elderly to pay attention to dietary combinations during food preparation, accelerating the popularization of nutritious diets among the elderly population (58).

Our research has several important implications for both study and education. First, this study provides a reference for research on nutrition and oxidative stress. There has been relatively little research in China on the relationship between nutrition and oxidative stress. Previous studies on the relationship between nutrients and oxidative stress levels have largely focused on animal experiments. Second, the findings of this study offer valuable insights for developing strategies to improve the diets of rural older adults. Due to a lack of dietary and nutritional knowledge among older adults in rural Northwest China, their eating habits are often unhealthy. Based on our findings, we can provide reasonable dietary guidance to older adults in rural areas. Finally, this study provides valuable guidance for the dietary management of non-communicable chronic diseases. Research shows that oxidative stress is related to the occurrence and development of chronic diseases. In this study, we further explored the relationship between different types of diets and oxidative stress biomarkers. This can provide dietary recommendations for older adults with chronic diseases.

This study has several limitations. First, because this is a cross-sectional study, we cannot determine a causal relationship between nutritional intake and oxidative stress. Second, the dietary data collected in this study were self-reported by the elderly, which introduces recall bias. Finally, this study was conducted within specific rural communities in two cities of Ningxia. Consequently, the sample may not be fully representative, and the findings may not be generalizable to broader populations. Additionally, our study only provides conclusions about the relationship between nutritional intake, 8-iso-PGF2α, and SOD. The relationship between other oxidative stress biomarkers and nutritional intake still needs further exploration by expanding the sample size.

## Conclusion

In summary, this study identified three distinct energy and nutrient intake profiles among rural older adults, which were significantly associated with variations in the oxidative stress biomarkers 8-iso-PGF2α and SOD. These findings underscore the substantial impact of nutrition on oxidative stress and provide a foundational reference for future research into dietary interventions for the aging population.

## Data Availability

The raw data supporting the conclusions of this article will be made available by the authors, without undue reservation.
