# Supplementary material for: Latent classes of energy and nutrient intake and their associations with oxidative stress in rural older adults: a cross-sectional study
Source: Front Nutr. 2025 Dec 8;12:1694444. doi: 10.3389/fnut.2025.1694444 (PMC12719268; doi:10.3389/fnut.2025.1694444)
Supplement: Supplementary file 1 [file Table_1.DOCX]

**Supplemental material A**

**Main Instruments and Equipment**

**Main Instruments and Equipment**

The main instruments and equipment used in this study are as follows.

AMR-100 Microplate Reader (Hangzhou Aoshen Co., Ltd.)

Ultrapure Water System (Sichuan Youpu Co., Ltd.)

Pipettes (20μL, 150μL, 100μL, 1000μL) (Sartorius AG, Germany)

L4 Visible Spectrophotometer (Shanghai Youke Co., Ltd.)

420-B Electric Constant Temperature Water Bath (Jiangsu Xinkang Co., Ltd.)

4°C Low-Temperature Refrigerator (Qingdao Haier Special Refrigeration Co., Ltd.)

-80°C Low-Temperature Freezer (Qingdao Haier Biomedical Co., Ltd.)

Centrifuge (Anhui Zhongke Zhongjia Scientific Instrument Co., Ltd.)

MW12-A Plate Washer (Shenzhen Mindray Bio-Medical Electronics Co., Ltd.)

**Main Reagents Source and Origin**

Malondialdehyde (MDA) Detection Kit (Nanjing Jianchen Bioengineering Institute)

Human 8-Isoprostane F2α (8-iso-PGF2α) ELISA Kit (Wuhan Yilai Ruit Biotechnology Co., Ltd.)

Superoxide Dismutase (SOD) Detection Kit (Nanjing Jianchen Bioengineering Institute)

Total Antioxidant Capacity (T-AOC) Detection Kit (Nanjing Jianchen Bioengineering Institute)

**Laboratory testing items**

**Serum MDA Concentration Detection**

1 Preparation

Strictly follow the reagent instructions to prepare Reagent Two Application Solution and Reagent Three Application Solution. The specific steps are as follows:

1. Sample Processing

Take out the samples to be tested and the detection reagents in advance, and allow them to equilibrate to room temperature for 20 minutes.

1. Prepare Reagent Two Application Solution

Add Reagent Two to 340 mL of distilled water and mix well, then store at 4°C.

c. Prepare Reagent Three Application Solution

Add Reagent Three to 64 mL of hot distilled water (90-100°C), cool, and then add 60 ml of glacial acetic acid. Dilute with 50% glacial acetic acid in a 2:1 ratio and store away from light.

2 Operational Process

1. MDA Operation Procedure

The MDA operation flow is shown in Table A1.

Table A1 MDA Sample Addition Operation Table

| Reagent Name | Blank Tube | Standard Tube | Test Tube | Control Tube |
| --- | --- | --- | --- | --- |
| 10 nmol/mL Standard (mL) | - | 0.1 | - | - |
| Anhydrous Ethanol (mL) | 0.1 | - | - | - |
| Test Sample (mL) | - | - | 0.1 | 0.1 |
| Reagent One (mL) | 0.1 | 0.1 | 0.1 | 0.1 |
| Mix (vortex mixer) | | | | |
| Reagent Two Application (mL) | 0.75 | 0.75 | 0.75 | 0.75 |
| Reagent Three Application (mL) | 0.75 | 0.75 | 0.75 | - |
| 50% Glacial Acetic Acid (mL) | - | - | - | 0.75 |

Note: “-” indicates that the reagent is not added.

b. MDA Detection Process

Sample Addition: Set the blank, control, test, and test blank tubes, and add samples according to Table A1.

Water Bath: Cover the centrifuge tubes, mix with a vortex mixer, secure with an explosion-proof clamp, and incubate in a water bath at 95°C for 40 minutes. Remove and cool under running water.

Centrifugation: Centrifuge at 3500 rpm for 10 minutes.

Measure Absorbance: Take the supernatant and transfer it to a cuvette. Measure the absorbance of each tube using a microplate reader at a wavelength of 532 nm.

3 Result Calculation

Calculate the results according to the MDA calculation formula.

**Serum 8-iso-PGF2α Concentration Detection**

1 Preparation

Strictly follow the reagent instructions to prepare the standard working solution, washing solution, biotinylated antibody working solution, and enzyme conjugate working solution. The specific methods are as follows:

a. Sample Processing

Take the sample out 20 minutes in advance to equilibrate to room temperature. Add 2 μL of the sample to 98 μL of sample diluent.

b. Prepare Standard Working Solution

Prepare the standard working solution at the following concentrations: 1000, 500, 250, 125, 62.5, 31.25, 15.63, 0 pg/mL.

1. Prepare Washing Solution

Heat in a 40°C water bath, dissolve, and then dilute with distilled water at a ratio of 1:24.

d. Prepare Biotinylated Antibody

Dilute the biotinylated antibody at a ratio of 1:99 with the diluent.

e. Prepare Enzyme Conjugate Working Solution

Dilute the enzyme conjugate at a ratio of 1:99 with the diluent.

2 Operational Process

Layout: Set blank, standard, and test holes separately; Sample addition: Add 50μL of standard and sample diluent to the blank hole, and 50μL of the sample to be tested in the remaining holes, then add 50μL of biotinylated antibody working solution to each hole; Water bath: Cover the enzyme-labeled plate and incubate in a 37℃ water bath for 45 minutes; Washing the plate: Discard the liquid in the holes, tap dry on clean absorbent paper, and wash the plate with a plate washer 3 times; Adding reagent: Add 100μL of enzyme conjugate working solution to each hole; Water bath: Cover the enzyme-labeled plate and incubate in a 37℃ water bath for 30 minutes; Washing the plate: Discard the liquid in the holes, tap dry on clean absorbent paper, and wash the plate with a plate washer 5 times; Adding reagent: Add 90μL of substrate solution (TMB) to each hole; Water bath: Cover the enzyme-labeled plate and incubate in the dark at 37℃ for about 15 minutes; Terminating the reaction: Add 50μL of stop solution to each hole to terminate the reaction; Measuring absorbance: Use the enzyme marker to measure the absorbance value in each hole at a wavelength of 450nm and save the reading results.

3 Result Calculation

Using the standard product absorbance value (OD value) as the x-coordinate and the standard product concentration value as the y-coordinate, plot the standard curve using Origin software. Then, substitute the sample OD value into the curve to calculate the concentration of 8-iso-PGF2α in the sample.

**Serum SOD Concentration Detection**

1 Preparation

The enzyme working solution and substrate application solution should be prepared separately in strict accordance with the requirements of the reagent instructions, with specific methods as follows.

1. Sample Processing

Remove the samples to be tested in advance, allow them to return to room temperature, then take 10uL of sample and add 40uL of distilled water to dilute 5 times;

1. Preparation of Enzyme Working Solution

The enzyme diluent is mixed uniformly in a 1:10 ratio to prepare the enzyme working solution;

1. Preparation of Substrate Application Solution

The buffer is mixed uniformly in a volume ratio of 1:200 to prepare the substrate application solution;

2 Operation Process

1. The SOD operation procedure is shown in Table A2.

Table A2 SOD Sample Addition Operation Table

| Reagent name | reference hole | Compare with the blank hole | Measurement hole | Determining the blank hole |
| --- | --- | --- | --- | --- |
| Sample to be tested (μL) | - | - | 20 | 20 |
| Distilled water (μL) | 20 | 20 | - | - |
| Enzyme working solution (μL) | 20 | - | 20 | - |
| Enzyme Dilution Solution (μL) | - | 20 | - | 20 |
| Substrate application liquid (μL) | 200 | 200 | 200 | 200 |

Note: “-” indicates that the reagent is not added.

b. SOD Detection Procedure

Sample Addition: Set up blank, control, test, and test blank tubes, and proceed with sample addition according to Table A2.

Water bath: 37°C water bath for 20 minutes.

Measure Absorbance: The microplate reader measures the absorbance of each well at a wavelength of 450nm.

3 Result Calculation

Calculate the results according to the SOD formula.

**Serum T-AOC Concentration Detection**

1 Preparation Work

Strictly follow the reagent instructions to prepare the standard working solution, Reagent 4 application solution, and ABTS working solution, as follows.

1. Specimen Preparation

Remove the sample to be tested and warm it to room temperature for 20 minutes.

1. Preparation of Standard Working Solution

Dilute the standard Trolox solution with distilled water to concentrations of 0.1, 0.2, 0.4, 0.8, and 1.0 mM.

1. Preparation of Reagent 4 Application Solution

Prepare Reagent 4 Application Solution at a ratio of 1:9:1:Enzyme Solution: Reagent 1. Prepare the solution immediately before use.

1. Preparation of ABTS Working Solution

Prepare the ABTS working solution at a ratio of 76:5:4:Reagent 1:Reagent 2:Reagent 3 application solution.

2 Operation Procedure

1. The T-AOC operation procedure is shown in Table A3.

Table A3 T-AOC Sample Addition Procedure

| Reagent Name | Blank Well | Standard Well | Assay Well |
| --- | --- | --- | --- |
| Distilled Water (μL) | 10 | － | － |
| Trolox Solutions of Different Concentrations (μL) | － | 10 | － |
| Test Sample (μL) | － | － | 10 |
| Reagent IV Application Solution (μL) | 20 | 20 | 20 |
| ABTS Working Solution (μL) | 170 | 170 | 170 |

Note: "-" indicates that the reagent is not added.

b. T-AOC Assay Procedure

Sample Addition: Set up blank, standard, and assay wells and add samples according to Table A3.

Water Bath: Incubate at room temperature for 6 minutes;

Absorbance Measurement: Measure the absorbance of each well at 405 nm using a microplate reader.

3 Calculation of Results

Draw a standard curve with the standard sample OD value as the horizontal axis and the standard sample concentration as the vertical axis. Use Excel to generate the curve formula. Substitute the OD values measured in the sample wells into the formula to calculate the results.
