# Supplementary material for: Latent classes of energy and nutrient intake and their associations with oxidative stress in rural older adults: a cross-sectional study
Source: Front Nutr. 2025 Dec 8;12:1694444. doi: 10.3389/fnut.2025.1694444 (PMC12719268; doi:10.3389/fnut.2025.1694444)
Supplement: Supplementary file 2 [file Table_2.DOCX]

**Supplemental material B**

TITLE:This is an example of LCA

DATA:

FILE IS C:\Users\kongweijuan\Desktop\mplus\nutrients\nutrients. Dat;

VARIABLE:

NAMES ARE number energy protein fat Carbon water meals cholesterol vitaminA vitaminB1 vitaminB2 vitaminC vitaminE calcium iron;

USEVARIABLES ARE energy protein fat Carbon water meals cholesterol vitaminA vitaminB1 vitaminB2 vitaminC vitaminE calcium iron;

Class=C(1);

Categorical=energy protein fat Carbon water meals cholesterol vitaminA vitaminB1 vitaminB2 vitaminC vitaminE calcium iron;

ANALYSIS:

TYPE=MIXTURE:START=1000 500;

SAVEDATA:FILE IS 101.txt;

SAVE IS CPROB:FORMAT IS FREE;

OUTPUT:TECH11 TECH14;

PLOT:TYPE IS PLOT3;

SERIES=energy protein fat Carbon water meals cholesterol vitaminA vitaminB1 vitaminB2 vitaminC vitaminE calcium iron(*).

Note. All five models have been fitted, and the 3-class parameter is optimal.
