# Supplementary material for: Latent classes of energy and nutrient intake and their associations with oxidative stress in rural older adults: a cross-sectional study
Source: Front Nutr. 2025 Dec 8;12:1694444. doi: 10.3389/fnut.2025.1694444 (PMC12719268; doi:10.3389/fnut.2025.1694444)
Supplement: Supplementary file 3 [file Table_3.DOCX]

**Supplemental material C**

Table C1 Reference intake of energy and major nutrients for the older people in the Dietary guide for elderly adults ^[1]^.

| Energy and nutrients | RNI ^a^ /AI ^b^ | | | | | | | |
| --- | --- | --- | --- | --- | --- | --- | --- | --- |
|  | 65~79 years old | | | | ≥ 80 years old | | | |
|  | Men | | Women | | Men | | Women | |
|  | Light ^c^ | | Moderate ^c^ | | Light ^c^ | | Moderate ^c^ | |
| Energy/(kcal/d) | 2050 | 2350 | 1700 | 1950 | 1900 | 2200 | 1500 | 1750 |
| Protein RNI/(g/d) | 20~30 | | | | | | | |
| Fat(%E ^d^) | <10 | | | | | | | |
| Carbohydrate(%E ^d^) | 50~65 | | | | | | | |
| Fiber(g/d) | 25 | | | | | | | |
| Cholesterol(mg/d) | ≤300 | | | | | | | |
| Vitamin A (μg RAE ^e^/d) | 800 | | 700 | | 800 | | 700 | |
| Vitamin B_1_RNI/(mg/d) | 1.4 | | 1.2 | | 1.4 | | 1.2 | |
| Vitamin B_2_RNI/(mg/d) | 1.4 | | 1.2 | | 1.4 | | 1.2 | |
| Vitamin C RNI/(mg/d) | 100 | | | | | | | |
| Vitamin E AI/(mgα-TE ^f^/d) | 14 | | | | | | | |
| Calcium RNI/(mg/d) | 1000 | | | | | | | |
| Iron RNI/(mg/d) | 12 | | | | | | | |

Note. RNI ^a^: Recommended intake; AI ^b^: Adequate Intake; ^c^ Physical activity level; ^d^ %E: The percentage of total energy; ^e^ Retinol activity equivalent (RAE, μg) =Dietary or supplement source all trans retinol（μg) +1/2 Supplement Pure All Trans β- Carotene（μg)+1/12 Dietary all trans β- Carotene（μg) +1/24 Other Dietary Vitamin A Procarotenoids（μg); ^f^α- Tocopherol equivalent（ α- TE, mg), total in diet α- TE equivalent (mg)=1 ×α- Tocopherol (mg)+0.5 ×β- Tocopherol (mg)+0.1 × γ – tocopherol (mg)+0.2 ×δ- Tocopherol (mg)+0.3 ×α- Triene tocopherol (mg)

Table C2 Nutrients intake grouping of older residents based on reference intake.

| Energy and nutrients | Men | | | | Women | | | |
| --- | --- | --- | --- | --- | --- | --- | --- | --- |
|  | RNI ^a^ /AI ^b^ | Nutrients intake ^c^ | Occupy RNI%/AI% | Group | RNI ^a^ /AI ^b^ | Nutrients intake ^c^ | Occupy RNI%/AI% | Group |
| Energy/(kcal/d) | 1900 | 1753.79±668.54 | <80,insufficiency  80~110, moderateness  >110, excessiveness | 1  2  3 | 1500 | 1576.83±586.92 | <80,insufficiency  80~110, moderateness  >110, excessiveness | 1  2  3 |
| Protein RNI/(g/d) | 65 | 54.28±27.98 |  |  | 55 | 45.78±24.62 |  |  |
| Fat(%E ^d^) | 42 | 36.52±3.31 |  |  | 33 | 33.14±20.72 |  |  |
| Carbohydrate(%E ^d^) | 238 | 224.15±87.39 |  |  | 188 | 205.35±76.48 |  |  |
| Vitamin A (μg RAE ^e^/d) | 800 | 420.64±198.90 | <80,insufficiency  80~110, moderateness  >110, excessiveness | 1  2  3 | 700 | 439.85±212.56 | <80,insufficiency  80~110, moderateness  >110, excessiveness | 1  2  3 |
| Vitamin C RNI/(mg/d) | 100 | 75.31±40.71 |  |  | 100 | 77.32±34.19 |  |  |
| Vitamin E AI/(mgα-TE ^f^/d) | 14 | 11.77±8.30 |  |  | 14 | 12.62±8.78 |  |  |

Note. Note. RNI ^a^: Recommended intake; AI ^b^: Adequate Intake; ^c^ Average intake; ^d^ %E: The percentage of total energy; ^e^ Retinol activity equivalent (RAE, μg) =Dietary or supplement source all trans retinol（μg) +1/2 Supplement Pure All Trans β- Carotene（μg)+1/12 Dietary all trans β- Carotene（μg) +1/24 Other Dietary Vitamin A Procarotenoids（μg); ^f^α- Tocopherol equivalent（α- TE, mg), total in diet α- TE equivalent (mg)=1 ×α- Tocopherol (mg)+0.5 ×β- Tocopherol (mg)+0.1 × γ – tocopherol (mg)+0.2 ×δ- Tocopherol (mg)+0.3 ×α- Triene tocopherol (mg); *Cholesterol intake should not be excessive, so only distinguishing between moderateness and excessiveness.

The participants of this study are all individuals with light physical activity.

**Reference**

1. China, N.H.C.o.t.P.s.R.o. Dietary guidance for the elderly. 2017. Available online: <http://www.nhc.gov.cn/ewebeditor/uploadfile/2018/06/20180613135619237.pdf>. (accessed on 20 August 2024).
